# The Histone Acetyltransferase CgHat1 Regulates Growth, Development, and Pathogenicity of *Colletotrichum gloeosporioides*

**DOI:** 10.3390/jof11110768

**Published:** 2025-10-24

**Authors:** Binghong Jia, Qian Zhou, Yiping Liu, Zhuohua Chen, Moxiang Liao, Chunsheng Zhu, Caisheng Xiao, Junwen Ai

**Affiliations:** 1 Institute of Cotton and Sericulture, Hunan Academy of Agricultural Sciences, Changsha 410127, China; srijbh@126.com (B.J.); zhouqian901010@163.com (Q.Z.); ypliu619@163.com (Y.L.); 18896954943@163.com (Z.C.); liaomx3098@126.com (M.L.); zhuchunsheng2002@163.com (C.Z.); 2 Yuelushan Laboratory, Changsha 410128, China

**Keywords:** histone acetyltransferase, pathogenicity, *C. gloeosporioides*

## Abstract

*Colletotrichum gloeosporioides* causes anthracnose on a wide range of plants, resulting in serious economic losses worldwide. However, the molecular mechanisms underlying its pathogenicity still remain largely unknown. In the past 20 years, the importance of acetylation/deacetylation modification in the pathogenicity of phytopathogens has already been highlighted, but how it functions in *C. gloeosporioides* is obscure. Here, we identified and functionally characterized a histone acetyltransferase CgHat1 in *C. gloeosporioides*. As suspected, CgHat1 is localized to the nucleus and regulates the acetylation levels of histone H4K5 and H4K12. Targeted gene deletion revealed that CgHat1 plays crucial roles in growth, colony pigmentation, and conidiation. Furthermore, we provided evidence showing that Δ*Cghat1* mutant is defective in conidial germination, appressorial formation, and response to endoplasmic reticulum (ER) stress. These combined effects lead to the decreased pathogenicity of Δ*Cghat1* mutant. Our studies not only firstly shed light on the pleiotropic roles of histone acetyltransferase in *C. gloeosporioides*, but also offer a potential fungicide target for anthracnose control.

## 1. Introduction

*Colletotrichum* spp. is one of the top 10 pathogens in plant-pathogenic fungi and almost every crop grown in the world is susceptible to one or more species of *Colletotrichum* [1,2]. Among them, *C. gloeosporioides* is one of the most common and widely distributed species in the world, which causes anthracnose on a wide range of plants, including *Gossypium hirsutum*, *Morus alba*, *Malus pumila*, and *Capsicum annuum*, resulting in economic losses of 30–60% or even more [3]. Apart from its economic importance, *C. gloeosporioides* has also emerged as a model for scientists to study fungal pathogenicity. For example, the actin-bundling protein CgFim1-organized F-actin dynamics during appressorium development regulates the pathogenicity of *C. gloeosporioides* in *Hevea brasiliensis* [4]. The conserved MAPK kinase CgMkk1 phosphorylates the transcriptional factor CgCrzA to respond to the CFW-induced stress and underpin the virulence of *C. gloeosporioides* in *Cunninghamia lanceolate* [5]. The secreted protein CgCsa-mediated Fe^3+^ homeostasis regulates the pathogenicity of *C. gloeosporioides* in *Capsicum* spp. [6]. Although much progress has been made in the study of its pathogenesis, the molecular mechanisms underlying pathogenicity still remain largely unknown.

The transcriptome analyses revealed that *Colletotrichum* spp. reprograms fungal gene expression to infect host plants [2]. The acetylation and deacetylation of histones are pivotal epigenetic mechanisms for reprograming gene transcription [7]. Histone acetylation promotes the opening of chromatin and the further binding of transcription factors, thus promoting gene transcription. Conversely, histone deacetylation reduces the accessibility of transcription factors by forming a closed chromatin conformation, thus inhibiting gene transcription [7,8]. The histone acetyltransferase Hat1 is one of the first identified histone acetyltransferases. It mainly promotes gene transcription by promoting the acetylation of lysine K5 and K12 in histone H4 [9,10].

Although the mechanism of Hat1 regulating histone acetylation is highly conserved, its biological function is species-specific. The ScHat1 in *Saccharomyces cerevisiae* is dispensable for growth, while the absence of *HAT1* gene in *Candida albicans* could cause elongated cells [11,12]. There are a few reports about the important roles of Hat1 on virulence in pathogenic fungi, but its pathogenic mechanism is different in different fungi. In the plant pathogenic fungus *Magnaporthe oryzae*, MoHat1 governs the pathogenicity mainly by regulating the turgor pressure of appressorium, while in the animal pathogenic fungus *Candida albicans*, CaHat1 affects the pathogenicity mainly by regulating the formation of infection structure [12,13]. These raise the question of how CgHat1 functions in *C. gloeosporioides*.

In the presented study, we identified the histone acetyltransferase CgHat1 in *C. gloeosporioides* and characterized its function. We revealed that nucleus-locating protein CgHat1 plays crucial roles in the acetylation of H4K5 and H4K12. Furthermore, we found that CgHat1 is involved in growth, colony pigmentation, and conidiation. Importantly, we provided evidence demonstrating the importance of this protein in responses to ER stress, appressorium development, and pathogenicity of *C. gloeosporioides*.

## 2. Materials and Methods

### 2.1. Strains and Culture Conditions

The *C. gloeosporioides* strain, isolated from *M. alba* leaves and identified by molecular and morphological analysis, was used as WT in this study and was preserved in Yuelushan Laboratory at −80 °C. All strains were cultured on CM, MM or PDA agar plates in darkness at 28 °C. Liquid CM was used to culture strains for extracting total proteins and harvesting conidia.

### 2.2. Targeted Gene Deletion, Complementation and Subcellular Localization

Targeted gene deletion was carried out by one-step gene replacement strategy [5]. First, two sequences of around 1.0 kb flanking the *CgHAT1* were amplified by PCR with primer pairs (Table S1). Then, the upstream and downstream flanking sequences were overlapped with the flanks of hygromycin resistance cassette (HPH), respectively. Finally, the resulting 3.4 kb fragments were introduced into the protoplasts of WT strain for gene deletion. For complementation assays, the *CgHAT1* gene and its native promoter regions were amplified by PCR with primer pairs (Table S1) using high-fidelity DNA polymerase and inserted into pYF11 vector. After sequencing, the construct was introduced into the protoplasts of Δ*Cghat1* mutant as described previously [14]. For the subcellular localization of CgHat1, the mycelia and conidia of the strains co-expressing CgHat1-GFP and H1-RFP were observed and visualized under a fluorescent microscope. The merged GFP fluorescence and RFP fluorescence indicated their co-localization in nucleus.

### 2.3. Growth and Stress Response Assays

To test hyphal growth, small agar blocks of the strains were cut from the periphery of the colonies and cultured on fresh CM, MM, and PDA agar plates at 28 °C in darkness. After 3 days incubation, the colony diameters were measured. To test stress response, the strains were cultured on fresh CM or CM supplemented with ER stress (2.5 mM DTT), osmotic stresses (1 M NaCl or 1 M KCl), cell wall integrity stresses (0.01% SDS or 400 μg/mL Congo Red), oxidative stress (5 mM H_2_O_2_), and rapamycin stress (25 nM rapamycin).

### 2.4. Conidiation, Appressorial Formation and Pathogenicity Assays

To test conidiation, the strains were first cultured in 200 mL liquid CM for 3 days and then the mycelia were filtered by three layers of lens paper, harvesting the conidia in filtrates for statistical analysis. The harvested conidia were also further washed by ddH_2_O twice and were resuspended to a concentration of 2 × 10^5^ spores per milliliter. Finally, resuspended conidia were inoculated on cover slips (hydrophobic) for conidial germination and appressorial formation. To test pathogenicity, the strains were inoculated onto the *M. alba* leaves in the high-humidity plates. After incubation in darkness at 28 °C for 3 days, the lesions were observed, photographed and assessed by ImageJ software [15].

### 2.5. Protein Extraction and Acetylation Analysis

The strains were cultured in liquid CM for 36 h and then the mycelia of each strain were harvested. Next, the mycelia were ground into fine powder in a mortar containing liquid nitrogen and resuspended with 1 mL RIPA (radio immunoprecipitation assay) buffer with protease inhibitors. The lysates were placed on the ice for 30 min and vortex-mixed once 10 min for protein lysing, followed by centrifugation at 13,400× *g* for 20 min at 4 °C to remove the cell debris. The supernatant lysates were collected as extracted proteins and were analyzed by 10% SDS-PAGE followed by Western blotting with anti-H4K5Ac (PTM BIO, Hangzhou, Zhejiang, China, PTM-119, 1:2000), anti-H4K12Ac (PTM BIO, Hangzhou, Zhejiang, China, PTM-121, 1:2000), anti-H4 (EpiZyme, Shanghai, Shanghai, China, P010074, 1:1000), and anti-Tubulin (Engibody, Dover, DE, USA, AT0003, 1:10,000) for detection.

### 2.6. Bioinformatics and Statistical Analysis

The phylogenetic tree of Hat1 proteins was constructed by MEGA5.05 using a neighbor-joining method with 1000 bootstrap replicates. The sequences of Hat1 proteins were collected from the NCBI database (https://www.ncbi.nlm.nih.gov/, accessed on 12 August 2025). The domain of CgHat1 were predicted using the SMART website (http://smart.embl-heidelberg.de/, accessed on 12 August 2025). For statistical analysis, all experiments were performed with at least three technical replicates and repeated three times, which showed consistent results. Data were assessed for normality and then analyzed by Student’s *t*-test with SPSS 20.0 software. *p* < 0.05 was considered as a significant difference.

## 3. Results

### 3.1. Identification and Bioinformatic Analysis of CgHat1

**Figure 1:**
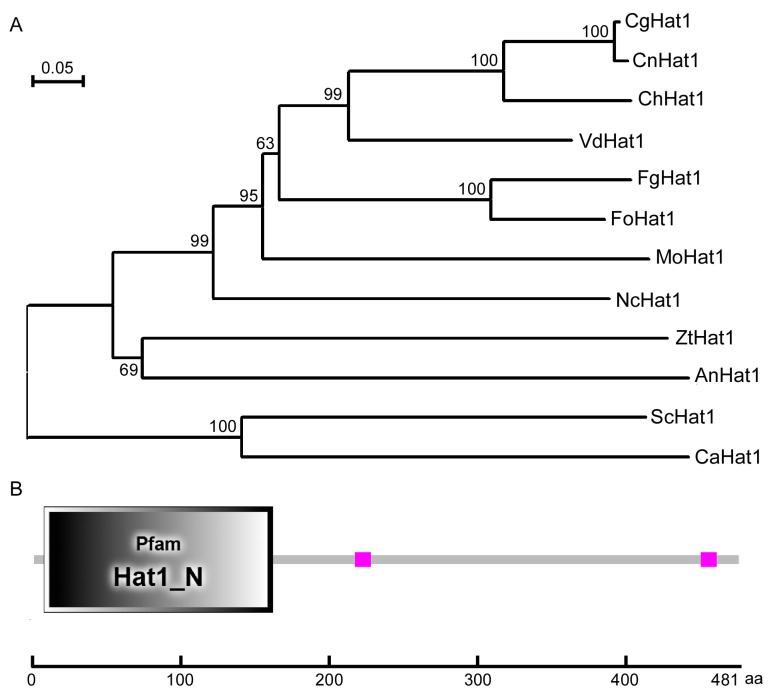
Phylogenetic analysis and domain prediction of CgHat1. (**A**). The phylogenetic tree of Hat1 proteins in diverse fungi constructed by MEGA5.05 using neighbor-joining method with 1000 bootstrap replicates. The GenBank accession numbers and the related organisms are as follows: CgHat1 (EQB43824.1, *Colletotrichum gloeosporioides*), CnHat1 (KAJ0285795.1, *Colletotrichum noveboracense*), ChHat1 (XP_018162470.1, *Colletotrichum higginsianum*), VdHat1 (KAF3354044.1, *Verticillium dahliae*), FgHat1 (XP_011326992.1, *Fusarium graminearum*), FoHat1 (KAI7768789.1, *Fusarium oxysporum*), MoHat1 (XP_003717199.1, *Magnaporthe oryzae*), NcHat1 (XP_957363.1, *Neurospora crassa*), ZtHat1 (XP_003854292.1, *Zymoseptoria tritici*), AnHat1 (XP_663818.1, *Aspergillus nidulans*), ScHat1 (NP_015324.1, *Saccharomyces cerevisiae*), and CaHat1 (KHC75880.1, *Candida albicans*). (**B**). The domain prediction of CfAda3 by the SMART website. The gray quadrilateral represents the Hat1 domain.

Using *S. cerevisiae* Hat1 sequence as a query, we acquired its homolog by a BLAST_P search in the *C. gloeosporioides* genome database and named it CgHat1. Phylogenetic analysis of CgHat1 and its homologs from other fungi revealed that Hat1 proteins are highly conserved, with MoHat1 being most homologous to Cnhat1 in *C. noveboracense* (98% identify and 99% similarity) and most distant to yeast Hat1p (still 30% identify and 47% similarity) (Figure 1A). CgHat1 encodes a 481-amino acid (aa) polypeptide and is predicted with a conserved N-terminal HAT domain (7–163 aa) by the SMART website (Figure 1B).

### 3.2. CgHat1 Is Localized to the Nucleus

**Figure 2:**
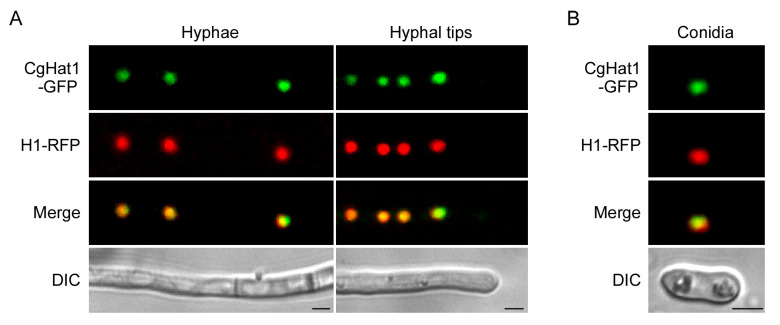
CgHat1 is localized to the nucleus. (**A**). CgHat1 is localized to the nucleus in hyphae. The hyphae of strains co-expressed CgHat1-GFP and H1-RFP observed under fluorescent microscope. The merged area of GFP and RFP signal showed that CgHat1-GFP localized in the nucleus. (**B**). CgHat1 is localized to the nucleus in conidia. The merged area showed that CgHat1-GFP localized in the nucleus. Bar = 5 μm.

To observe the subcellular localization of CgHat1, a green fluorescent protein (GFP) tag was fused to the C-terminus of CgHat1. Spot green fluorescent signals were observed under fluorescent microscope. To test whether the spot signals indicate nucleus, a nucleus marker H1-RFP was introduced into the CgHat1-GFP expressed strains. We found that all the green fluorescence was co-localized with green fluorescence in mid and tip regions of the vegetative hyphae (Figure 2A). Furthermore, we also found that CgHat1-GFP was co-localized well with H1-RFP in conidia (Figure 2B). These results indicate that CgHat1 is localized to the nucleus in both vegetative hyphae and conidia.

### 3.3. CgHat1 Regulates the Acetylation of H4K5 and H4K12

**Figure 3:**
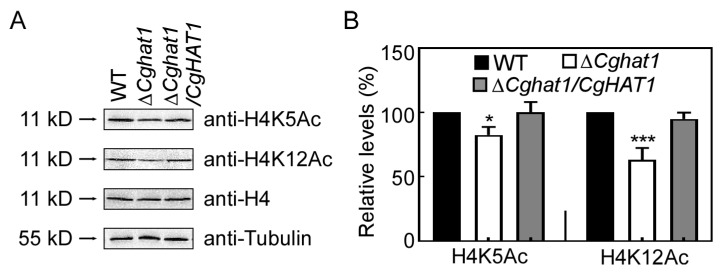
CgHat1 regulates the acetylation of H4K5 and H4K12. (**A**). Western blot analysis of the H4K5 and H4K12 acetylation with anti-H4K5, anti-H4K12, anti-H4, and anti-Tubulin antibodies. (**B**). The relative levels of acetylation were calculated as the anti-H4K5Ac/anti-H4 and anti-H4K12Ac/anti-H4 ratios by integrated signal density using ImageJ 1.48v software. The ratio of WT was normalized as 1 (* *p* < 0.05, *** *p* < 0.001).

To test the roles of CgHat1 in nucleus, we obtained the *CgHAT1* gene deletion mutant Δ*Cghat1* according to the homologous recombination principle (Figure S1A,B). Since two mutant strains were indistinguishable in general phenotypes, we randomly selected Δ*Cghat1#*15 for further study. The yeast ScHat1 acetylates the histone H4K5 and H4K12 [9,10]. Thus, we examined the acetylation levels of H4K5 and H4K12. Compared to wide-type (WT) and complemented strain Δ*Cghat1/CgHAT1*, Δ*Cghat1* showed significantly decreased acetylation levels of both H4K5 and H4K12 (Figure 3). These results suggest that CgHat1 regulates the acetylation of H4K5 and H4K12.

### 3.4. CgHat1 Is Important for the Growth on PDA Media and for the Colony Pigmentation

**Figure 4:**
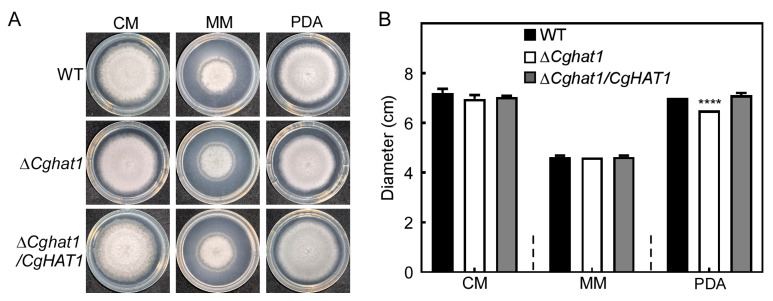
CgHat1 is involved in the growth on PDA media. (**A**). Colony morphology of the WT, Δ*Cghat1*, and Δ*Cghat1/CgHAT1* strains on the top of CM, MM, and PDA agar plates. (**B**). Statistical analysis of the colony diameters of strains on CM, MM, and PDA media. Error bars represent SD and asterisks denote statistical significances (**** *p* < 0.0001).

**Figure 5:**
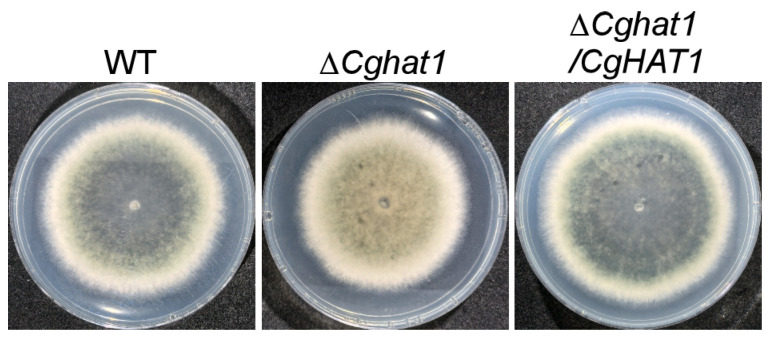
CgHat1 is important for colony pigmentation. Colony morphology of the WT, Δ*Cghat1*, and Δ*Cghat1/CgHAT1* strains on the bottom of PDA agar plates.

To address the role of CgHat1 in growth, the WT, Δ*Cghat1*, and Δ*Cghat1/CgHAT1* strains were cultured on CM, MM, and PDA agar plates. The results showed that all the strains exhibit similar growth levels on CM and MM agar plates, whereas the Δ*Cghat1* mutant showed significantly decreased growth rates compared to WT and Δ*Cghat1/CgHAT1* on PDA agar plates (Figure 4). Additionally, compared to the colony pigmentation in WT and Δ*Cghat1/CgHAT1*, the Δ*Cghat1* mutant showed absolutely no pigmentation (Figure 5). These results suggest that CgHat1 plays important in growth on PDA media and in colony pigmentation.

### 3.5. CgHat1 Is Critical for Asexual Development

**Figure 6:**
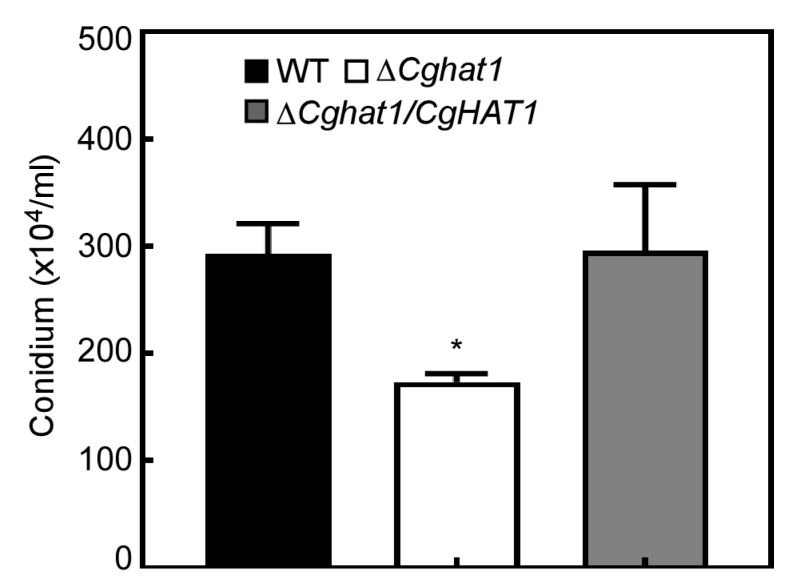
CgHat1 is critical for asexual development. The conidia produced by WT, Δ*Cghat1*, and Δ*Cghat1/CgHAT1* strains were collected, quantified, and analyzed (* *p* < 0.05). Three biological experiments were performed for each strain with three technical replicates per biological experiment.

As asexual conidia play an important role in the disease cycle and infection of *Colletotrichum* [16,17], the conidiation production of the WT, Δ*Cghat1*, and Δ*Cghat1/CgHAT1* strains were collected, quantified, and analyzed. After 3 days of incubation, the WT and Δ*Cghat1/CgHAT1* strains produced about 300 × 10^4^ conidia per milliliter, compared to no more than 200 × 10^4^ conidia/mL produced by Δ*Cghat1* mutant (Figure 6). This result indicates that CgHat1 is critical for asexual development.

### 3.6. CgHat1 Is Required for Full Virulence

**Figure 7:**
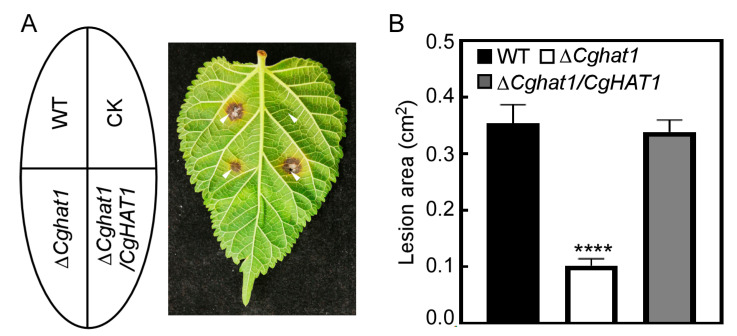
CgHat1 is required for full virulence. (**A**). The leaves of *M. alba* inoculated with WT, Δ*Cghat1*, and Δ*Cghat1/CgHAT1* strains. Photographs taken at 3 days post-inoculation (dpi). The arrowheads denote infected sites. (**B**). Statistical analysis of lesion areas calculated by Image J on *M. alba* leaves. Error bars represent SD and asterisks denote significant differences (**** *p* < 0.0001).

As a phytopathogen, we focus more of our interest on examining the roles of CgHat1 in virulence. The WT, Δ*Cghat1*, and Δ*Cghat1/CgHAT1* strains were inoculated onto the *M. alba* leaves. After 4 days incubation, the lesions caused by WT and Δ*Cghat1/CgHAT1* strains showed obviously larger than that caused by Δ*Cghat1* mutant (Figure 7A). Furthermore, the statistical analysis revealed that the WT and Δ*Cghat1/CgHAT1* caused more than 0.3 cm^2^ lesion areas on *M. alba* leaves, compared to about 0.1 cm^2^ lesion areas caused by Δ*Cghat1* mutant (Figure 7B). This result indicates that CgHat1 is required for full virulence of *C. gloeosporioides*.

### 3.7. CgHat1 Plays Important Roles in Conidial Germination and Appressorial Formation

**Figure 8:**
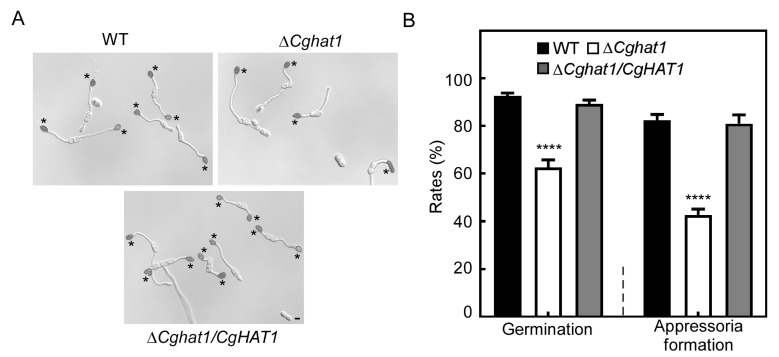
CgHat1 plays important roles in conidial germination and appressorial formation. (**A**) The conidia of WT, Δ*Cghat1*, and Δ*Cghat1/CgHAT1* strains incubated on artificial hydrophobic surfaces, and observed under microscopy. Asterisks indicate appressoria. Bar = 5 μm. (**B**). Statistical analysis of the conidial germination and appressorial formation rates. Error bars represent SD and asterisks denote significant differences (**** *p* < 0.0001).

To investigate the possible reasons for the decreased virulence in Δ*Cghat1* mutant, we tested the appressorial development. The conidia of WT, Δ*Cghat1*, and Δ*Cghat1/CgHAT1* strains were incubated on artificial hydrophobic surfaces. The percentages of conidial germination were more than 85% in WT and Δ*Cghat1/CgHAT1*, compared to about 60% of that in the Δ*Cghat1* mutant (Figure 8). Similar decreased rates of Δ*Cghat1* mutant were also observed in appressorial formation, compared to WT and Δ*Cghat1/CgHAT1* strains (Figure 8). This result indicates that CgHat1 plays important roles in conidial germination and appressorial formation.

### 3.8. CgHat1 Regulates the Response to ER Stress

**Figure 9:**
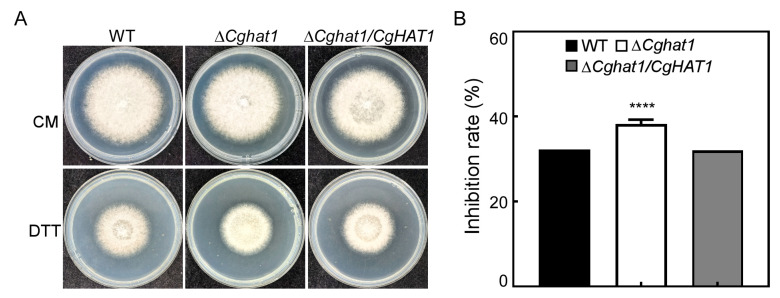
CgHat1 regulates the response to ER stress. (**A**). Colony morphology of the WT, Δ*Cghat1*, and Δ*Cghat1/CgHAT1* strains on the CM and CM supplemented with 2.5 mM DTT agar plates. (**B**). Statistical analysis of inhibition rates of the strains to DTT stress. Error bars represent SD and asterisks denote statistical significances (**** *p* < 0.0001).

In order to grow normally and infect plants, pathogenic fungi must overcome many stresses [18,19]. Therefore, we tested whether CgHat1 may also associate with the response to environmental stresses. We found that the Δ*Cghat1* mutant showed significantly higher inhibition rates to endoplasmic reticulum (ER) stress (DTT) than that in WT and Δ*Cghat1/CgHAT1* strains (Figure 9). Meanwhile, we also found that the Δ*Cghat1* mutant showed similar sensitivity with WT and Δ*Cghat1/CgHAT1* strains to osmotic stresses (NaCl and KCl), cell wall integrity stresses (SDS and CR), oxidative stress (H_2_O_2_) and rapamycin stress (Figure S2). These results suggest that CgHat1 regulates the response to ER stress.

## 4. Discussion

In the long-term and complicated arms race with host plants, pathogens, including *Colletotrichum* spp., successfully infect host plants through various strategies such as gene transcription rearrangement [2]. HATs are necessary co-activators for transcriptional activation by modifying histones [20,21]. In the past 20 years, the importance of HATs in the pathogenicity of fungi have already been highlighted [22,23,24]. The Hat1 protein is one of the first identified histone acetyltransferases and mainly promotes gene transcription by promoting the acetylation of lysine K5 and K12 in histone H4 [9,10], which is consistent with our study showing that CgHat1 is localized in nucleus to regulate the H4K5Ac and H4K12Ac in *C. gloeosporioides*. Furthermore, the phylogenetic analysis revealed that the sequence of Hat1 proteins is highly similar in fungi, especially in *Colletotrichum* species. The conserved role of Hat1 in acetylation and sequence similarity of such proteins might forecast its conservative biological functions in different organisms.

Actually, CgHat1 plays important roles in conidiation, which is consistent with its homologues in *Pestalotiopsis microspora* [25]. Meanwhile, its biological function might also be species-specific. Our results revealed that CgHat1 is dispensable for growth in synthetic medium and positively regulates growth in semisynthetic medium. However, the yeast ScHat1 is not involved in growth, while the absence of the *HAT1* gene in *C. albicans* could cause elongated cells [11,12]. Furthermore, although both of the Hat1 proteins in *M. oryzae* and *C. albicans* participate in the pathogenic process, their pathogenic mechanisms vary across different species. To achieve successful infection, *M. oryzae* employs Hat1 to regulate the turgor pressure of appressorium, whereas *C. albicans* primarily utilizes Hat1 to influence pathogenicity by controlling the formation of its infection structures [12,13]. These raise the question of how CgHat1 functions in the pathogenicity of *C. gloeosporioides*.

The significantly decreased virulence of Δ*Cghat1* revealed that CgHat1 is required for pathogenicity. We reasoned that the defect in virulence of Δ*Cghat1* mutant was directly due to the decreased conidial germination and appressorial formation, which is essential for the penetration and host-colonization of *Colletotrichum* spp. [16,17]. Moreover, the studies in *M. oryzae* showed that the host-derived ER stress is a common barrier for the successful infection of pathogens [26,27]. Therefore, the susceptibility of Δ*Cghat1* mutant to ER stress might also be a reason for its pathogenicity defect. Additionally, the melanin layer is essential for the penetration and pathogenicity of *M. oryzae* and *Alternaria alternata* [28,29,30]. The reduced pigmentation in Δ*Cghat1* mutant might be another reason for its reduced pathogenicity. However, it is unclear whether there is a direct correlation between pigmentation and pathogenicity in *Colletotrichum* and further studies are highly warranted.

## 5. Conclusions

Taken together, we identified and functional characterized the histone acetyltransferase CgHat1 in *C. gloeosporioides*. Our findings showed that CgHat1 is localized in the nucleus and regulates the H4K5Ac and H4K12Ac. The Δ*Cghat1* mutant is pleiotropic in defects, ranging from growth to conidiation and responses to ER stress. Our studies provide new insight for exploring such a protein as a potential fungicide target for anthracnose control. However, the genes regulated by CgHat1 are not yet clear in *Colletotrichum*. Further studies will focus on the interactive analysis between transcriptome and acetylomics.

## Data Availability

The original contributions presented in this study are included in the article/Supplementary Materials. Further inquiries can be directed to the corresponding authors.

## Supplementary Materials

The following supporting information can be downloaded at https://www.mdpi.com/article/10.3390/jof11110768/s1: Figure S1: Targeted gene deletion of *CgHAT1* gene in *C. gloeosporioides*; Figure S2: CgHat1 is dispensable for the response to multiple stresses; Table S1: Primers used in this study.

## Abbreviations

The following abbreviations are used in this manuscript:

CM	Complete medium
CR	Congo red
DTT	Dimethyl sulfoxide
ER	Endoplasmic reticulum
HAT	Histone acetyltransferase
MM	Minimal medium
PCR	Polymerase Chain Reaction
PDA	Potato Dextrose Agar
