# Supplementary material for: The Histone Acetyltransferase CgHat1 Regulates Growth, Development, and Pathogenicity of Colletotrichum gloeosporioides
Source: J Fungi (Basel). 2025 Oct 24;11(11):768. doi: 10.3390/jof11110768 (PMC12653700; doi:10.3390/jof11110768)
Supplement: Supplementary file 1 [file jof-11-00768-s001.zip › Figure S1.pdf]

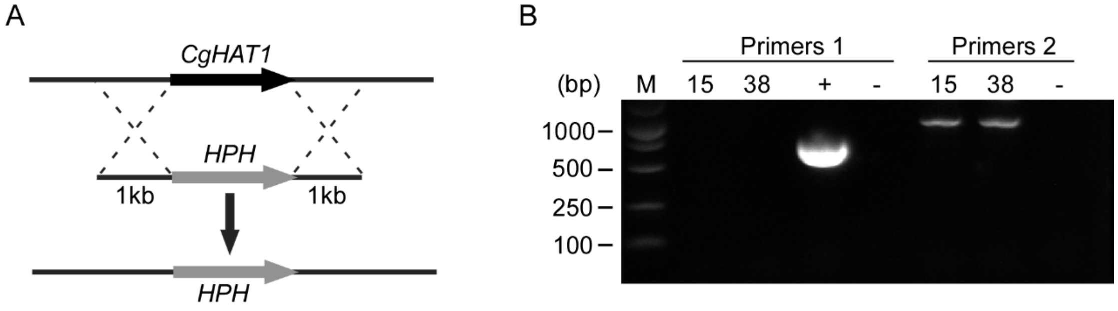

Figure S1 Targeted gene deletion of *CgHAT1* gene in *C. gloeosporioides*.

A. Schematic illustration for *CgHAT1* targeted gene deletion. B. The Δ*Cghat1* mutants were validated with primer pairs 1 (*HAT1*-3F/*HAT1*-3R) and primer pairs 2 (*HAT1*-4F/HPHR). M: marker. 15 and 38 indicate different Δ*Cghat1* mutants, respectively. +: WT; -: negative control.
