# Supplementary material for: The Histone Acetyltransferase CgHat1 Regulates Growth, Development, and Pathogenicity of Colletotrichum gloeosporioides
Source: J Fungi (Basel). 2025 Oct 24;11(11):768. doi: 10.3390/jof11110768 (PMC12653700; doi:10.3390/jof11110768)
Supplement: Supplementary file 1 [file jof-11-00768-s001.zip › Figure S2.pdf]

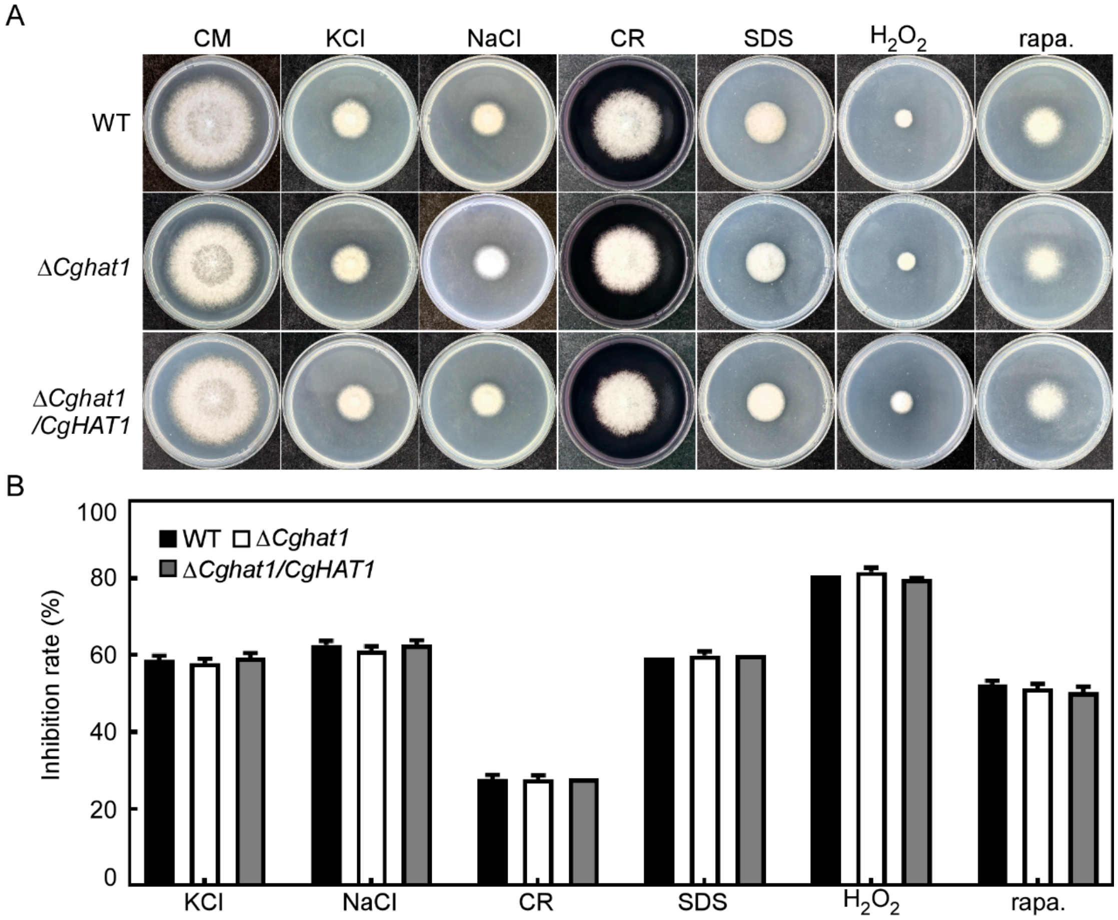

Figure S2 CgHat1 is dispensable for the response to multiple stresses.

A. Colony morphology of the WT, Δ*Cghat1*, and Δ*Cghat1/CgHAT1* strains on the CM and CM supplemented with osmotic stresses (NaCl and KCl), cell wall integrity stresses (SDS and CR), oxidative stress (H_2O_2) and rapamycin stress. B. Statistical analysis of inhibition rates of the strains to various stresses. Error bars represent SD.
