# Supplementary material for: The Histone Acetyltransferase CgHat1 Regulates Growth, Development, and Pathogenicity of Colletotrichum gloeosporioides
Source: J Fungi (Basel). 2025 Oct 24;11(11):768. doi: 10.3390/jof11110768 (PMC12653700; doi:10.3390/jof11110768)
Supplement: Supplementary file 1 [file jof-11-00768-s001.zip › Table S1.pdf]

Table S1 Primers used in this study

Primer name	Sequence (5'-3')	Remark
HAT1-5F	ACTCACTATAGGGCGAATTGGGTACTCAAATTGGTTTCGGCACCTCGGCTTGTTT	Construction of CgHat1-GFP
HAT1-5R	CACCACCCCGGTGAACAGCTCCTCGCCCTTGCTCACGGCGTCTTCGATCCTGACCTT	Construction of CgHat1-GFP
HAT1-1F	CCAGAATACCAACACCAACGCTT	amplify Cg*HAT1* 5' flank sequence
HAT1-1R	TTGACCTCCACTAGCTCCAGCCAAGCCGGTGTCGACAGTGAAGGCAGG	amplify Cg*HAT1* 5' flank sequence
HAT1-2F	CAAAGGAATAGAGTAGATGCCGACCGGCTCTCCACAGCTTACGACGAT	amplify Cg*HAT1* 3' flank sequence
HAT1-2R	GCAAAGAAGGTCAAGAAGGCT	amplify Cg*HAT1* 3' flank sequence
HAT1-3F	ACACCATCCTACACTTTCGTCG	validation of Cg*HAT1* deletion
HAT1-3R	GCACCAAGTCTATGTTTCTCCC	validation of Cg*HAT1* deletion
HAT1-4F	GCTTGTTTCTCCTCGCCGTGCTT	validation of Cg*HAT1* deletion
HPHR	CGCTACTGCTACAAGTGGGGCT	validation of Cg*HAT1* deletion
